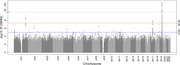# Multimodal Genetic Analysis of Brain Amyloidosis

**DOI:** 10.1002/alz.090566

**Published:** 2025-01-03

**Authors:** Ting‐Chen Wang, Derek B. Archer, Muhammad Ali, Yiyang Wu, Elizabeth Mormino, Rachel F. Buckley, Annie J. Lee, Andrew J. Saykin, Philip L. De Jager, Julie A. Schneider, David A. Bennett, Lisa L. Barnes, Badri N. Vardarajan, Richard Mayeux, Brian W. Kunkle, William S. Bush, C. Dirk Keene, Sudha Seshadri, Reisa A Sperling, Prashanthi Vemuri, Vijay K. Ramanan, Gerard D. Schellenberg, Matthew J. Huentelman, Kara L. Hamilton‐Nelson, Margaret A. Pericak‐Vance, Alison M. Goate, Jonathan L. Haines, Thomas J. Montine, Gary W Beecham, Carlos Cruchaga, Timothy J. Hohman, Logan C. Dumitrescu, ADNI, Knight Alzheimer Disease Research Center (Knight ADRC), the Dominantly Inherited Alzheimer Network (DIAN), ADNI‐DOD, the Australian Imaging Biomarkers, Lifestyle (AIBL) Study, A4 Study Team, Alzheimer’s Disease Sequencing Project (ADSP)

**Affiliations:** ^1^ Vanderbilt Genetics Institute, Vanderbilt University Medical Center, Nashville, TN USA; ^2^ Vanderbilt Memory and Alzheimer’s Center, Vanderbilt University Medical Center, Nashville, TN USA; ^3^ Vanderbilt Genetics Institute, Institute for Medicine and Public Health Vanderbilt University Medical Center, Nashville, TN USA; ^4^ Department of Neurology, Vanderbilt University Medical Center, Nashville, TN USA; ^5^ Department of Psychiatry, Washington University, St. Louis, MO USA; ^6^ NeuroGenomics and Informatics Center, Washington University, St. Louis, MO USA; ^7^ Department of Neurology and Neurological Sciences, Stanford University School of Medicine, Stanford, CA USA; ^8^ Department of Neurology, Harvard Medical School, Boston, MA USA; ^9^ Brigham and Women’s Hospital and Department of Neurology, Massachusetts General Hospital, Harvard Medical School, Boston, MA USA; ^10^ Department of Neurology, Columbia University Medical Center, New York, NY USA; ^11^ Taub Institute for Research on Alzheimer’s Disease and The Aging Brain, Columbia University Medical Center, New York, NY USA; ^12^ Department of Neurology, The New York Presbyterian Hospital, New York, NY USA; ^13^ Department of Radiology and Imaging Sciences, Center for Neuroimaging, School of Medicine, Indiana University, Indianapolis, IN USA; ^14^ Department of Medical and Molecular Genetics, School of Medicine, Indiana University, Indianapolis, IN USA; ^15^ Cell Circuits Program, Broad Institute, Cambridge, MA USA; ^16^ Department of Neurology, Center for Translational and Computational Neuroimmunology, Columbia University Medical Center, New York, NY USA; ^17^ Rush Alzheimer’s Disease Center, Rush University Medical Center, Chicago, IL USA; ^18^ Rush Alzheimer's Disease Center, Rush University Medical Center, Chicago, IL USA; ^19^ The Institute for Genomic Medicine, Columbia University Medical Center, New York, NY USA; ^20^ Taub Institute for Research on Alzheimer’s Disease and the Aging Brain, Columbia University Medical Center, New York, NY USA; ^21^ John P. Hussman Institute for Human Genomics, University of Miami Miller School of Medicine, Miami, FL USA; ^22^ Department of Population and Quantitative Health Sciences, Institute for Computational Biology, Case Western Reserve University, Cleveland, OH USA; ^23^ Department of Laboratory Medicine and Pathology, University of Washington, Seattle, WA USA; ^24^ Boston University School of Medicine, Boston, MA USA; ^25^ Framingham Heart Study, Framingham, MA USA; ^26^ Department of Neurology, Massachusetts General Hospital, Harvard Medical School, Boston, MA USA; ^27^ Mayo Clinic, Rochester, MN USA; ^28^ Department of Neurology, Mayo Clinic, Rochester, MN USA; ^29^ Department of Pathology and Laboratory Medicine, University of Pennsylvania Perelman School of Medicine, Philadelphia, PA USA; ^30^ Neurogenomics Division, Translational Genomics Research Institute, Phoenix, AZ USA; ^31^ Ronald M Loeb Center for Alzheimer’s Disease, Department of Genetics & Genomic Sciences, Icahn School of Medicine at Mount Sinai, New York, NY USA; ^32^ Department of Pathology, Stanford University School of Medicine, Stanford, CA USA; ^33^ John T. MacDonald Foundation Department of Human Genetics, University of Miami, Miami, FL USA; ^34^ NeuroGenomics & Informatics Center, Washington University School of Medicine, St. Louis, MO USA; ^35^ Department of Psychiatry, Washington University School of Medicine, St. Louis, MO USA; ^36^ Hope Center for Neurological Disorders, Washington University School of Medicine, St. Louis, MO USA; ^37^ Knight Alzheimer’s Disease Research Center, Washington University, St Louis, MO USA; ^38^ Department of Genetics, Washington University School of Medicine, St Louis, TN USA

## Abstract

**Background:**

Genome‐wide association studies (GWAS) in Alzheimer’s disease (AD) leveraging endophenotypes beyond case/control diagnosis, such as brain amyloid β pathology, have shown promise in identifying novel variants and understanding their potential functional impact. In this study, we leverage two brain amyloid β pathology measurement modalities, PET imaging and neuropathology, to address sample size limitations and to discover novel genetic drivers of disease.

**Method:**

We conducted a meta‐analysis on an amyloid PET imaging GWAS (N = 7,036, 35% amyloid positive, 53.67% female, age = 71) and an autopsy GWAS of brain amyloidosis (N = 6,519, 63.08% amyloid positive, 51.34% female, age at death = 83), both adjusted for covariates including age, sex and principal components of genetic ancestry. All participants in both GWAS were unrelated individuals of European descent. We defined amyloid positivity as moderate or frequent neuritic plaques using the Consortium to Establish a Registry for Alzheimer’s Disease (CERAD) staging scores within each autopsy cohort. A Gaussian mixture model (GMM) was applied to each amyloid PET imaging cohort to identify the cohort and tracer‐specific cut‐offs that differentiate amyloid positive and negative populations.

**Result:**

The genome‐wide significant results from the meta‐analysis identified three known AD loci (*APOE*, *CR1*, and *BIN1*) and a novel chromosome 17 locus (rs35635959, intergenic, MAF = 0.27, OR = 1.18, p = 1.47 × 10^‐8^). The MAGMA gene‐level analysis excluding the *APOE* region suggests significant associations between rs35635959 and *COASY*, *PLEKHH3*, and *TUBG2* on chromosome 17, implying the potential roles of these genes on amyloidosis. Functional annotations of rs35635959 leveraging brain eQTL databases indicate its association with differential gene expression of *COASY* and *TUBG2* in AD brains. ROSMAP bulk and single‐nucleus RNAseq analyses link *TUBG2* downregulation to the higher amyloid burden and AD diagnosis while suggesting such observations are enriched in excitatory and inhibitory neurons, reaching FDR significance.

**Conclusion:**

This study is the most extensive European GWAS of brain amyloidosis, and our findings replicate known AD loci and identify a possible novel locus (index SNP rs35635959) on chromosome 17. Functional annotations of the novel variant indicate *TUBG2*, implicating in microtubule organization, warrants further assessment. Ongoing efforts aim to validate these novel effects using independent datasets.